# Treatment of elderly patients with non-ST-elevation myocardial infarction: the nationwide POPular age registry

**DOI:** 10.1007/s12471-023-01812-0

**Published:** 2023-09-28

**Authors:** Marieke E. Gimbel, Dean R. P. P. Chan Pin Yin, Wout W. A. van den Broek, Renicus S. Hermanides, Floris Kauer, Annerieke H. Tavenier, Dirk Schellings, Stijn L. Brinckman, Salem H. K. The, Martin G. Stoel, Ton A. C. M. Heestermans, Saman Rasoul, Mireille E. Emans, Machiel van de Wetering, Paul F. M. M. van Bergen, Ronald Walhout, Debby Nicastia, Ismail Aksoy, Arnoud van ’t Hof, Paul Knaapen, Cees-Joost Botman, Anho Liem, Cornelis de Nooijer, Joyce Peper, Johannes C. Kelder, Jurriën M. ten Berg

**Affiliations:** 1 Department of Cardiology, St. Antonius Hospital, Nieuwegein, The Netherlands; 2 Department of Cardiology, Isala Hospitals, Zwolle, The Netherlands; 3 Department of Cardiology, Albert Schweitzer Hospital, Dordrecht, The Netherlands; 4 Department of Cardiology, Slingeland Hospital, Doetinchem, The Netherlands; 5 Department of Cardiology, Tergooi, Blaricum, The Netherlands; 6 Department of Cardiology, Treant Hospitals, Emmen, The Netherlands; 7 Department of Cardiology, Medisch Spectrum Twente, Enschede, The Netherlands; 8 Department of Cardiology, Noord-west Hospital Group, Alkmaar, The Netherlands; 9 Department of Cardiology, Zuyderland Medical Centre, Heerlen, The Netherlands; 10 Department of Cardiology, Ikazia Hospital, Rotterdam, The Netherlands; 11 Department of Cardiology, BovenIJ Hospital, Amsterdam, The Netherlands; 12 Department of Cardiology, Dijklander Hospital, Hoorn, The Netherlands; 13 Department of Cardiology, Gelderse Vallei Hospital, Ede, The Netherlands; 14 Department of Cardiology, Gelre Hospitals, Apeldoorn, The Netherlands; 15 Department of Cardiology, Admiraal de Ruyter Hospital, Goes, The Netherlands; 16 Department of Cardiology, Maastricht University Medical Centre and , Cardiovascular Research Institute Maastricht (CARIM), Maastricht, The Netherlands; 17 Department of Cardiology, Amsterdam University Medical Centre, Amsterdam, The Netherlands; 18 Department of Cardiology, Catharina Hospital, Eindhoven, The Netherlands; 19 Department of Cardiology, Franciscus Gasthuis, Rotterdam, The Netherlands; 20 Department of Cardiology, Máxima Medical Centre, Veldhoven, The Netherlands

**Keywords:** Antiplatelet therapy, Non-ST-elevation myocardial infarction, Elderly, Therapy

## Abstract

**Objective:**

We describe the current treatment of elderly patients with non-ST-elevation myocardial infarction (NSTEMI) enrolled in a national registry.

**Methods:**

The POPular AGE registry is a prospective, multicentre study of patients ≥ 75 years of age presenting with NSTEMI, performed in the Netherlands. Management was at the discretion of the treating physician. Cardiovascular events consisted of cardiovascular death, myocardial infarction and ischaemic stroke. Bleeding was classified according to the Bleeding Academic Research Consortium (BARC) criteria.

**Results:**

A total of 646 patients were enrolled between August 2016 and May 2018. Median age was 81 (IQR 77–84) years and 58% were male. Overall, 75% underwent coronary angiography, 40% percutaneous coronary intervention, and 11% coronary artery bypass grafting, while 49.8% received pharmacological therapy only. At discharge, dual antiplatelet therapy (aspirin and P2Y_12_ inhibitor) was prescribed to 56.7%, and 27.4% received oral anticoagulation plus at least one antiplatelet agent. At 1‑year follow-up, cardiovascular death, myocardial infarction or stroke had occurred in 13.6% and major bleeding (BARC 3 and 5) in 3.9% of patients. The risk of both cardiovascular events and major bleeding was highest during the 1st month. However, cardiovascular risk was three times as high as bleeding risk in this elderly population, both after 1 month and after 1 year.

**Conclusions:**

In this national registry of elderly patients with NSTEMI, the majority are treated according to current European Society of Cardiology guidelines. Both the cardiovascular and bleeding risk are highest during the 1st month after NSTEMI. However, the cardiovascular risk was three times as high as the bleeding risk.

**Supplementary Information:**

The online version of this article (10.1007/s12471-023-01812-0) contains supplementary material, which is available to authorized users.

## What’s new?

- Coronary angiography was performed in a high proportion of elderly patients with non-ST-elevation myocardial infarction (75%), but only 40% of the patients underwent percutaneous coronary intervention and 11% coronary artery bypass graft.
- In these elderly patients, preference was given more often to clopidogrel over the more potent P2Y_12_ inhibitors.
- The risk of stent thrombosis and target vessel revascularisation was very low.
- The cardiovascular risk was three times as high as the bleeding risk.

## Introduction

An increasingly large proportion of patients presenting with non-ST-elevation myocardial infarction (NSTEMI) are elderly. According to the European Society of Cardiology (ESC) guidelines on the management of acute coronary syndromes (ACS) in patients presenting without persistent ST-segment elevation and on dual antiplatelet therapy (DAPT), patients with NSTEMI are treated with DAPT consisting of aspirin and one of the more potent P2Y_12_ inhibitors, ticagrelor or prasugrel [1, 2]. The choice of the more potent P2Y_12_ inhibitors is based on the pivotal Trial to assess Improvement in Therapeutic Outcomes by optimising platelet InhibitioN with prasugrel Thrombolysis In Myocardial Infarction 38 (TRITON-TIMI 38) [3] and PLATelet inhibition and patient Outcomes (PLATO) [4] trial comparing prasugrel or ticagrelor, respectively, with clopidogrel in patients with ACS. However, elderly patients were under-represented in these trials and risk assessment for choosing the optimal antithrombotic treatment in elderly patients is more complicated than in younger patients, because they are at higher risk for both ischaemic and bleeding events [5, 6]. In daily practice, elderly patients are less likely to receive guideline-recommended treatment, such as undergoing coronary angiography, compared to younger patients [7–10]. In this registry study we describe the contemporary treatment of elderly patients (≥ 75 years of age) admitted to Dutch hospitals and medical centres with NSTEMI. We observe whether elderly patients are treated according to the guidelines and present both cardiovascular and bleeding outcomes until 1 year after NSTEMI.

## Methods

### Study design and population

The POPular AGE registry is an investigator-initiated, prospective, observational, multicentre study of patients aged ≥ 75 years presenting with type I NSTEMI. Patients were recruited between 1 August 2016 and 7 May 2018 at 21 sites in the Netherlands. Three of these institutions were also participating in the POPular AGE trial [11], a randomised controlled trial comparing clopidogrel to ticagrelor in patients aged 70 years or older with NSTE-ACS. Patients included in a randomised controlled trial (including the POPular AGE trial) were excluded from this registry.

Decisions regarding medical therapy, performing coronary angiography and, if necessary, subsequent percutaneous coronary intervention (PCI) or coronary artery bypass grafting (CABG), were at the discretion of the attending physicians. Physicians were asked to calculate the GRACE and CRUSADE risk scores with the aim of being able to describe the study population. The use of risk scores to adjust antiplatelet therapy was not demanded by the protocol. This study was conducted according to the principles of the Declaration of Helsinki and was approved by the local Medical Research Ethics Committee. All patients provided written informed consent. If patients died before informed consent could be obtained, data were collected anonymously if there was no indication that patients would have declined if they had still been alive.

### Data collection

Information was extracted from the patients’ electronic medical records or retrieved from the patients’ general practitioner. In addition, patients were sent a questionnaire after 1 month and after 12 months inquiring about current medication use and new hospital stays. If a patient reported having had an event, the necessary documentation was collected and reviewed. Total duration of different antithrombotic strategies and reasons for P2Y_12_ inhibitor cessation were collected using the electronic medical records of both the hospital and the general practitioner.

### Outcomes and definitions

Cardiovascular events consisted of cardiovascular death, non-fatal myocardial infarction and non-fatal ischaemic stroke at 1‑year follow-up. Also, all-cause death, stent thrombosis, target vessel revascularisation (TVR), rehospitalisation for unstable angina, transient ischaemic attack (TIA) and any bleeding requiring medical attention classified according to the BARC bleeding criteria [12] at 1‑year follow-up were captured. Myocardial infarction was defined according to the fourth universal definition [13]. Stroke was defined as an acute new neurological deficit ending in death or lasting > 24 h and not attributable to another identifiable cause. TIA was defined as an acute new neurological deficit lasting < 24 h and not attributable to another identifiable cause, and stent thrombosis was classified according to the Academic Research Consortium criteria [14].

Antithrombotic therapy at discharge consisted of either aspirin, a P2Y_12_ inhibitor and/or oral anticoagulation (OAC). Treatment was further classified as monotherapy, DAPT (aspirin plus a P2Y_12_ inhibitor), dual therapy (OAC combined with aspirin or a P2Y_12_ inhibitor) or triple therapy (OAC, aspirin and a P2Y_12_ inhibitor).

### Statistical analysis

Missing data were assumed to be missing at random. Multiple imputation by predictive mean matching combined using Rubin’s rule was used to impute missing data. Continuous variables are presented as mean ± standard deviation (SD) unless the distribution was skewed, in which case variables are presented as median and interquartile range (IQR), and categorical variables are presented as frequency and percentage. All outcomes were calculated using Kaplan-Meier estimates. All tests were two-tailed and a *p*-value < 0.05 was used to characterise statistical significance. Kaplan-Meier curves were used to illustrate occurrence of cardiovascular events and BARC 3 or 5 bleeding over time. We performed one subgroup analysis comparing pharmacologically treated patients with patients who were treated by either PCI or CABG. We performed a univariate analysis of variables known to be predictors for cardiovascular events or bleeding [15–18]: age, renal function, haemoglobin level at admission, previous medical history of stroke, PCI, CABG, myocardial infarction, diabetes mellitus or peripheral arterial disease, use of OAC and current smoker. Variables with a *p*-value < 0.100 were included in the multivariate analysis to correct for confounders. All analyses were performed using IBM SPSS Statistics version 24 (IBM Corp., Armonk, NY, USA) and R statistical software version 3.4.2.

## Results

### Baseline characteristics

A total of 646 patients were enrolled in the study. Baseline characteristics are presented in Tab. 1 and Table S1 (Electronic Supplementary Material). Median age was 81 (IQR 77–84) years and 58% were male (*n* = 372). The previous medical history of the patients showed in 31% myocardial infarction (*n* = 201), in 15% CABG (*n* = 98) and in 18% atrial fibrillation (*n* = 118; Tab. 1). Almost 75% of the patients had a high GRACE risk score (> 140) (*n* = 449) and 34% of the patients had a high CRUSADE bleeding score (> 40) (*n* = 220; Electronic Supplementary Material). Overall, the proportion of missing data was low (< 6%), except for left ventricular function (in 28% of the patients), GRACE risk score (20%) and discharge destination (29%; Electronic Supplementary Material).

**Table 1:** Baseline characteristics

	*n* = 646
Age (years), median (IQR)	81 (77–84)
Male	57.6 (372/646)
Weight < 60 kg	8.0 (52/646)
*Previous medical history*	
Myocardial infarction	31.1 (201/646)
PCI	29.7 (192/646)
CABG	15.2 (98/646)
Stroke	7.3 (47/646)
Heart failure	6.5 (42/646)
Atrial fibrillation	18.3 (118/646)
Hypertension	66.4 (429/646)
Dyslipidaemia	45.2 (292/646)
Diabetes mellitus	27.2 (176/646)
Current smoker	7.0 (42/604)
Family history	19.5 (126/646)
*During hospital stay*	
CAG	74.5 (481/646)
No coronary artery disease	9.5 (45/476)
1‑vessel disease	26.5 (126/476)
2‑vessel disease	26.7 (127/476)
3‑vessel disease	35.9 (171/476)
Graft dysfunction	5.3 (25/476)
PCI	39.8 (257/646)
CABG	11.0 (71/646)
Pharmacological therapy only	49.8 (322/646)
*At discharge*	
Aspirin	77.8 (485/623)
Clopidogrel	49.9 (311/623)
Ticagrelor	33.1 (206/623)
NOAC	11.2 (70/623)
VKA	20.2 (126/623)

All numbers are percentages unless stated otherwise

*IQR* interquartile range, *PCI* percutaneous coronary intervention, *CABG* coronary artery bypass grafting, *CAG* coronary angiography, *NOAC* non-vitamin‑K oral anticoagulant, *VKA* vitamin K antagonist

### Treatment

During their hospital stay, 75% of patients underwent coronary angiography (*n* = 481), 88% of whom had a significant coronary lesion (*n* = 424) (defined as: left main-stenosis of ≥ 50% and ≥ 70% at another location). PCI was performed in 40% of patients (*n* = 257), 11% underwent CABG (*n* = 71), and 50% received pharmacological treatment only (*n* = 322). The Appendix in the Electronic Supplementary Material contains additional data regarding the non-revascularised patients. At discharge, 56.7% of the patients received DAPT (*n* = 353), 17.3% received dual therapy (*n* = 108) and 10.1% were discharged on triple therapy (*n* = 63) (Tab. 2). Antiplatelet therapy in combination with OAC consisted mostly of clopidogrel, with only 4% of patients receiving ticagrelor. Eighty-four percent of patients received either DAPT, dual therapy or triple therapy at discharge (*n* = 524). Patients who did not undergo coronary angiography were less often treated with triple therapy or DAPT (Tab. 2). Antiplatelet therapy was discontinued most frequently within the 1st month: for aspirin in 3.4% of the patients and for the P2Y_12_ inhibitor in 4.2%. Reasons to discontinue the P2Y_12_ inhibitor were peri-operative discontinuation to undergo CABG (18.5%), bleeding (16.7%), and revision of diagnosis (14.8%).

**Table 2:** Antithrombotic regimen for the total population with known antithrombotic therapy at discharge, stratified for patients who underwent coronary angiography (*CAG*) during hospital admission and those who did not

	Total population (*n* = 623)	No CAG (*n* = 165)	CAG (*n* = 471)	*p*-value
Triple therapy	10.1 (63/623)	3.9 (6/152)	12.1 (57/471)	0.004
DAPT	56.7 (353/623)	47.4 (72/152)	59.7 (281/471)	0.008
P2Y_12_ inhibitor + (N)OAC	14.4 (90/623)	25.7 (39/152)	10.8 (51/471)	< 0.001
Aspirin + (N)OAC	2.9 (18/623)	1.3 (2/152)	3.4 (16/471)	0.183
Monotherapy aspirin	8.2 (51/623)	11.2 (17/152)	7.2 (34/471)	0.121
Monotherapy P2Y_12_ inhibitor	2.1 (13/623)	2.0 (3/152)	2.1 (10/471)	0.911
Monotherapy (N)OAC	3.9 (24/623)	5.3 (8/152)	3.4 (16/471)	0.299
No antithrombotic therapy	1.8 (11/623)	3.3 (5/152)	1.3 (6/471)	0.101

All numbers are percentages; Triple therapy consists of aspirin, a P2Y_12_ inhibitor and a (non-vitamin-K) oral anticoagulant

*DAPT* dual antiplatelet therapy consisting of aspirin and a P2Y_12_ inhibitor, (*N)OAC* (non-vitamin-K) oral anticoagulant

### Outcomes

**Fig. 1:**
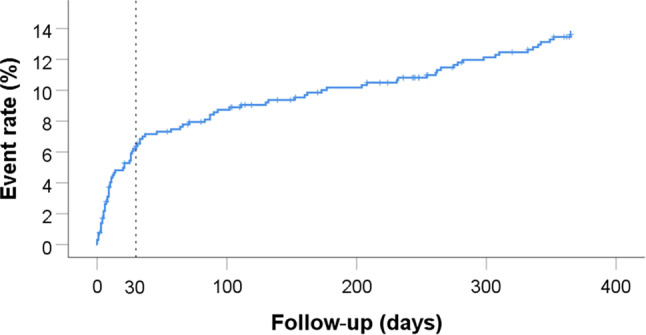
Kaplan-Meier curve of the primary endpoints cardiovascular (*CV*) death, myocardial infarction (*MI*) and stroke

**Fig. 2:**
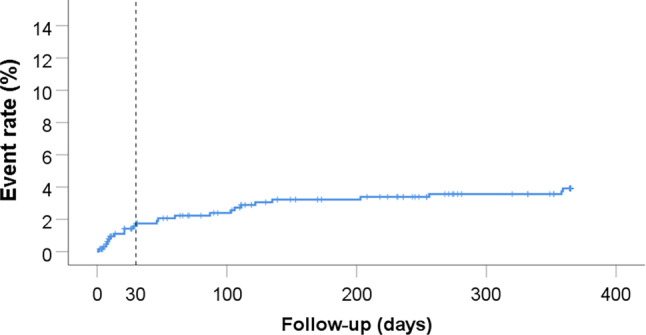
Kaplan-Meier curve of major bleeding not related to coronary artery bypass grafting. (*BARC* Bleeding Academic Research Consortium)

CV death, myocardial infarction or stroke had occurred in 6.4% (*n* = 41) of patients after 1 month and in 13.6% (*n* = 86) after 1 year (Tab. 3, Fig. 1). While the risk of cardiovascular events was highest during the 1st month, it persisted until the end of follow-up, as illustrated by the Kaplan-Meier curve. Recurrent myocardial infarction had occurred in 6.5% (*n* = 40) after 1 year; only one patient had a stent thrombosis (incidence 1/257; 0.4%). Major bleeding (defined as BARC 3 or 5) had occurred in 1.7% (*n* = 11) of patients after 1 month and in 3.9% (*n* = 24) after 1 year (Tab. 3). Also, the risk for major bleeding was highest in the 1st month (Fig. 2). Of note is that the cardiovascular risk was three times as high as the bleeding risk in this elderly population, both after 1 month and after 1 year.

**Table 3:** Outcomes

	30-day FU *n* = 646	1‑year FU *n* = 646
CV death, MI, stroke	6.4	13.6
All-cause death	4.6	13.0
CV death	3.4	6.3
MI	1.9	6.5
ACS	3.5	8.7
Definite stent thrombosis	0	0
Probable stent thrombosis	0.2	0.2
TVR	0.6	1.8
Stroke	0.8	2.0
TIA	0.2	0.7
BARC 2	2.5	9.1
BARC 3–5	3.3	5.6
BARC 3 and 5	1.7	3.9
BARC 2, 3 and 5	5.4	12.0
Discontinuing aspirin	3.4	8.7
Discontinuing P2Y_12_ inhibitor	4.2	8.8

All numbers are percentages

*FU* follow-up, *CV* cardiovascular, *MI* myocardial infarction, *ACS* acute coronary syndrome, *TVR* target vessel revascularisation, *TIA* transient ischaemic attack, *BARC* Bleeding Academic Research Consortium

A subanalysis comparing pharmacologically treated patients (*n* = 322) with patients who were treated by either PCI or CABG (*n* = 324) showed an event rate (CV death, myocardial infarction, or stroke) of 18.7% vs 8.7% (*p* = 0.034, adjusted hazard ratio 1.66 [95% confidence interval 1.04–2.65]) at 1 year. The outcome was corrected for age, previous medical history of CABG, previous medical history of stroke and estimated glomerular filtration rate. Also, the rate of BARC 3 or 5 bleeding was slightly higher although not significantly different (4.8% vs 3.1%, *p* = 0.454; adjusted hazard ratio 1.37 [95% confidence interval 0.60–3.11]) at 1 year in the pharmacologically treated group. This outcome was corrected for previous medical history of PCI and haemoglobin level at admission.

### Discussion

In this large national registry of patients aged 75 years or older with NSTEMI we observed, firstly, that this group of patients is characterised by a high ischaemic risk illustrated by frequent previous myocardial infarction, PCI and CABG. Second, compared to the SWEDEHEART registry of NSTEMI patients, and in accordance with the ESC guideline [1], coronary angiography was performed in a high percentage of cases (75%), but only 40% of the patients underwent PCI, while CABG was performed in 11%. Third, preference was given more often to clopidogrel over the more potent P2Y_12_ inhibitors. Fourth, the risk for cardiovascular and bleeding events was highest in the 1st month after hospital admission. However, the cardiovascular risk was three times as high as the bleeding risk in this elderly population, both after 1 month and after 1 year. Fifth, despite extensive coronary artery disease the risk of stent thrombosis and TVR was very low.

Compared to the nationwide SWEDEHEART registry of NSTEMI patients, in our registry the use of DAPT as well as that of aspirin and the more potent P2Y_12_ inhibitors was lower [19]. In contrast, in SWEDEHEART, aspirin use (78% vs 90%) [19] and use of the more potent P2Y_12_ inhibitors (33% vs 61%) was higher than in the START antiplatelet Italian registry [10]. These differences are very likely due to a higher concomitant use of OAC in our registry (31% vs 6.5% in SWEDEHEART) and the ESC guideline advice not to combine the stronger P2Y_12_ inhibitors with oral anticoagulants. Regarding interventions, a comparable percentage of patients underwent coronary angiography [19]. In our registry, a surprising low percentage of patients underwent PCI and this was lower than in SWEDEHEART (40% vs 53%) [19] and the POPular AGE trial [11], where PCI was performed in 47% of patients. In our registry, 11% of the patients underwent CABG, which is comparable to SWEDEHEART and the POPular AGE trial. The low number of our patients undergoing PCI is noteworthy, taking into consideration the very high percentage of patients (88% of patients undergoing coronary angiography) in whom a severe coronary artery lesion was identified. Another ACS registry reports a similar number of conservatively managed elderly patients (54%) [20]. Also, in the After Eighty study, a randomised controlled trial investigating invasive versus conservative treatment in patients aged 80 years or older, 51% of patients randomised to invasive treatment were not revascularised [21]. The relatively low number of patients who undergo revascularisation may indicate that a conservative strategy is often preferred because elderly patients are considered to be at too high a risk of complications if treated invasively, rather than a lack of adherence to the guidelines.

In our registry, we observed that both the risk of bleeding and the risk of thrombosis was highest in the 1st month. However, the cardiovascular risk was three times as high as the bleeding risk in this elderly population, both after 1 month and after 1 year. A similar risk pattern was seen in the subanalysis of elderly patients in TRITON-TIMI 38 [3], while the subanalysis of elderly patients in the PLATO [22] and POPular AGE [11] trials showed a cardiovascular risk about twice as high as the bleeding risk. In our cohort, the relatively low bleeding risk may be explained in part by the discontinuation of triple therapy after 30 days (10.1% of the study population used triple therapy). Also, it is likely that patients who developed moderate or severe bleeding discontinued at least one antithrombotic agent. Undoubtedly, this reduces the bleeding risk but may lead to an increased residual cardiovascular risk. In addition, we observed that the risk of stent thrombosis and TVR was very low, indicating that most of the cardiovascular risk is not stent related but more likely caused by multivessel disease.

The POPular Age registry is a large representative cohort study of elderly patients with NSTEMI in the Netherlands. Patients were included from academic as well as large and small non-academic centres. We had only a small amount of missing data, enabling us to perform a reliable evaluation of the current treatment of these elderly patients.

However, there were some limitations to our study. First, inherent to the observational design, it can be assumed that selection by indication occurred; therefore comparisons between treatment regimens could not be made. Second, although this is a large registry, the occurrence of major bleeding was low, with only 24 events. Finally, missing data might have biased the results, but this risk was minimised by using multiple imputation.

In conclusion, we report that elderly patients aged 75 years or older with NSTEMI are often treated according to current guidelines. Although the percentage of patients undergoing coronary angiography is similar to that in younger patients, elderly patients undergo PCI less often and are frequently treated conservatively. Also, elderly patients are more often discharged with clopidogrel instead of the more potent P2Y_12_ inhibitors. In this registry, the risk of both cardiovascular and bleeding events was highest in the 1st month after NSTEMI. However, the cardiovascular risk was three times as high as the bleeding risk in this elderly population, both after 1 month and after 1 year.

### Supplementary Information

Additional supplementary information on baseline characteristics and treatment
