# Supplementary material for: Treatment of elderly patients with non-ST-elevation myocardial infarction: the nationwide POPular age registry
Source: Neth Heart J. 2023 Sep 28;32(2):84–90. doi: 10.1007/s12471-023-01812-0 (PMC10834918; doi:10.1007/s12471-023-01812-0)
Supplement: Supplementary file 1 — Additional supplementary information on baseline characteristics and treatment [file 12471_2023_1812_MOESM1_ESM.docx]

Supplementary appendix

Table S1. Supplementary baseline characteristics

|  | N = 646 |
| --- | --- |
| BMI – mean ± SD | 26.9 ± 4.2 |
| Medical history |  |
| PAD | 12.5 (81/646) |
| COPD | 12.2 (79/646) |
| LVEF <30%^*^ | 4.8 (21/433) |
| At admission |  |
| Haemoglobin (mmol/L) – median, IQR | 8.3 (7.6-9.0) |
| Haematocrit (L/L) – median, IQR | 0.40 (0.37-0.43) |
| eGFR (ml/min/1.73m^2^) – median, IQR | 60.6 (45.7-74.2) |
| eGFR < 60 (ml/min/1.73m^2^) | 49.5 (320/646) |
| CK max (U/L) – median, IQR | 170 (96.3-367) |
| Killip class I | 82.8 (535/646) |
| Killip class II | 12.4 (80/646) |
| Killip class III | - 1. (30/646) |
| Killip class IV | 0.2 (1/646) |
| GRACE score > 140^#^ | 74.2 (449/605) |
| CRUSADE score > 40 | 34.1 (220/645) |
| During hospital stay |  |
| CAG | 74.5 (481/646) |
| Radial access site | 76.3 (367/481) |
| Femoral access site | 15.0 (97/481) |
| At discharge |  |
| Beta-blocker | 77.7 (484/623) |
| ACE-inhibitor | 47.5 (296/623) |
| Angiotensin II inhibitor | 23.4 (146/623) |
| Calcium antagonist | 29.9 (186/623) |
| Cholesterol inhibitor | 85.4 (532/623) |
| Diuretics | 34.8 (217/623) |
| Anti-diabetics | 23.9 (149/623) |
| Aldosterone antagonist | 11.1 (69/623) |
| PPI | 86.8 (541/623) |
| Left ventricular ejection fraction >50% | 57.5 (264/459) |
| Left ventricular ejection fraction 30-50% | 34.9 (160/459) |
| Left ventricular ejection fraction <30% | 7.6 (35/459) |
| Diagnosis Unstable angina | 3.9 (25/646) |
| Diagnosis NSTEMI | 87.9 (568/646) |
| Discharge destination home^‡^ | 87.3 (475/544) |
| Discharge destination rehabilitation^‡^ | 1.8 (10/544) |
| Discharge destination nursing home^‡^ | 2.6 (14/544) |
| Discharge destination other department in hospital^‡^ | 4.8 (26/544) |
| Discharge destination^‡^  Deceased | 3.5 (19/544) |

All numbers are percentages unless stated otherwise. IQR interquartile range, BMI body mass index, SD standard deviation, kg kilogram, PAD peripheral arterial disease, COPD chronic obstructive pulmonary disease, LVEF left ventricular ejection fraction, eGFR estimated glomerular filtration rate (CKD-EPI formula), CK creatine-kinase, CAG coronary angiography, ACE-inhibitor angiotensin-converting-enzyme inhibitor, PPI proton pump inhibitor, NSTEMI non-ST-elevation myocardial infarction. *33% missing, #20% missing, ‡ 29% missing.

Detailed information regarding patients who underwent coronary angiography without revascularisation

A total of 165 patients underwent coronary angiography but did not undergo revascularisation. The degree of vessel disease was missing in 3 patients. Of those 162 patients:

- 45 patients had no significant coronary artery disease
- 32 patients had one-vessel disease
- 32 patients had two-vessel disease
- 53 patients had three-vessel disease
